# Supplementary material for: Anti-Diabetic Activity of Glycyrrhetinic Acid Derivatives FC-114 and FC-122: Scale-Up, In Silico, In Vitro, and In Vivo Studies
Source: Int J Mol Sci. 2023 Aug 15;24(16):12812. doi: 10.3390/ijms241612812 (PMC10454726; doi:10.3390/ijms241612812)
Supplement: Supplementary file 1 [file ijms-24-12812-s001.zip › ijms-2506683-supplementary.pdf]

Supplementary data

Anti-Diabetic Activity of Glycyrrhetinic Acid Derivatives FC-114 and FC-122: Scale-Up, In Silico, In Vitro, and In Vivo Studies

Samuel Álvarez–Almazán[1], Luz Cassandra Solís–Domínguez[1#], Paulina Duperou–Luna[2#], Teresa Fuerte–Gómez[1#], Martin González–Andrade[3], María E. Aranda-Barradas[1], Juan Francisco Palacios–Espinosa[2], Jaime Pérez–Villanueva[2], Félix Matadamas–Martínez[2,‡], Susana Patricia Miranda–Castro[1], Crisoforo Mercado–Marquez[4], and Francisco Cortés–Benítez [2,*]

[1] Laboratory of Biotechnology, Unidad de Posgrado, Facultad de Estudios Superiores Cuautitlán Campus 1, Universidad Nacional Autónoma de México, State of Mexico 54740, Mexico; samuel.alvarez@cuautitlan.unam.mx (S.Á.-A.); cassandrasolis433@gmail.com (L.C.S.-D.); teresafuerte52@gmail.com (T.F.-G.); mariaaranda500@gmail.com (M.E.A.-B.); spmcunam55@gmail.com (S.P.M.-C.)

[2] Laboratory of Synthesis and Isolation of Bioactive Substances, Departamento de Sistemas Biológicos, División de Ciencias Biológicas y de la Salud, Universidad Autónoma Metropolitana–Xochimilco (UAM–X), Mexico City 04960, Mexico; duper_pau@icloud.com (P.D.-L.); jpalacios@correo.xoc.uam.mx (J.F.P.-E.); jpvillanueva@correo.xoc.uam.mx (J.P.-V.); felixmatadamas@yahoo.com.mx (F.M.-M.)

[3] Laboratory of Biosensors and Molecular Modelling, Departamento de Bioquímica, Facultad de Medicina, Universidad Nacional Autónoma de México, Mexico City 04510, Mexico; martin@bq.unam.mx

[4] Isolation and Animal Facility Unit, Facultad de Estudios Superiores Cuautitlán 28, Universidad Nacional Autónoma de México, State of Mexico 54714, Mexico; crisofo@cuautitlan.unam.mx

* Correspondence: jcortesb@correo.xoc.uam.mx; Tel.: +52-55-5483-4000 (ext. 7259)

† These authors contributed equally to this work.

‡ Current Address: Medical Research Unit in Infectious and Parasitic Diseases, UMAE Hospital de Pediatría, Centro Médico Nacional Siglo XXI, Instituto Mexicano del Seguro Social, Mexico City 06720, Mexico.

Images of Molecular Docking validation. Per-residue contacts charts of FC–122 and acarbose during 200–ns Molecular dynamics simulations for the α–glucosidase. Institutional Review Board of Facultad de Estudios Superiores Cuautitlán (protocol code C23_02 on March 2023).

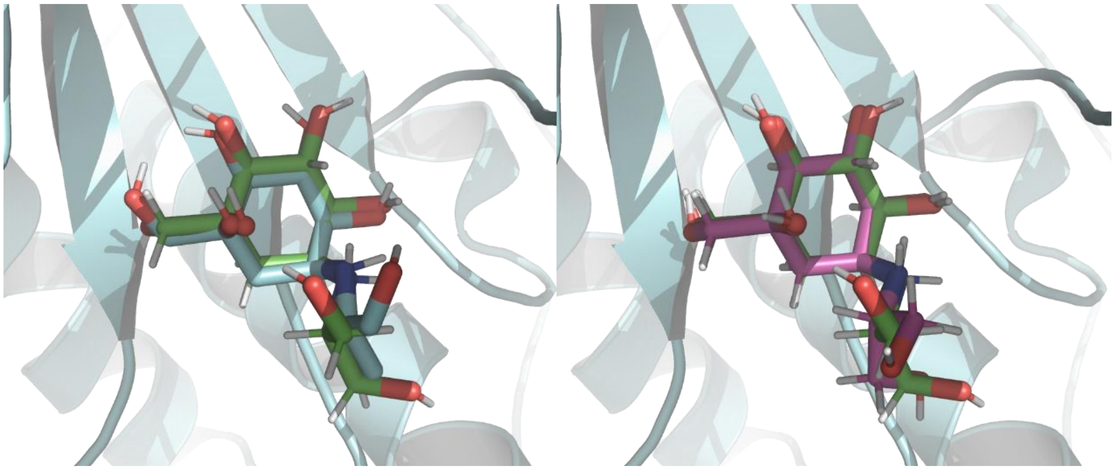

Figure S1. Binding pose of re–docked VOG structure using Autodock (cyan) and GOLD (violet) onto the native pose of VOG (green).

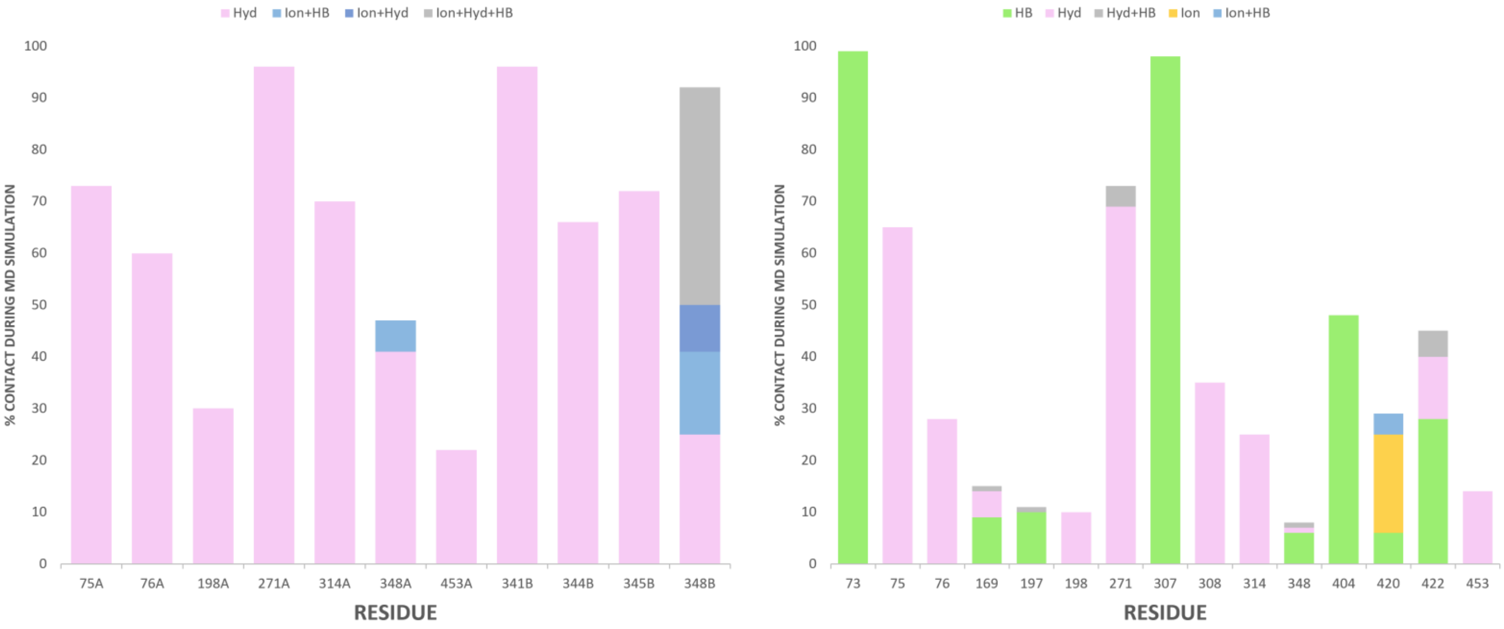

Figure S2. Per-residue contacts with FC–122 (left) and acarbose (right) during 200–ns Molecular dynamics (MD) simulations for the α–glucosidase. Three types of contacts are shown: Hydrogen bonds (green), hydrophobic contacts (pink), and ionic interactions (yellow). Also, other colors can show up if a particular residue is involved in multiple types of contact with the ligand.
